# Universal testing for hepatitis B and hepatitis C in the emergency department: a cost-effectiveness and budget impact analysis of two urban hospitals in the United Kingdom

**DOI:** 10.1186/s12962-022-00388-7

**Published:** 2022-11-14

**Authors:** Jack Williams, Peter Vickerman, Elizabeth Smout, Emma E. Page, Khine Phyu, Mark Aldersley, Gaia Nebbia, Sam Douthwaite, Laura Hunter, Murad Ruf, Alec Miners

**Affiliations:** 1 Department of Health Services Research and Policy, London School of Hygiene and Tropical Medicine, 15-17 Tavistock place, London, WC1H 9SH UK; 2 The National Institute for Health Research Health Protection Research Unit (NIHR HPRU) in Blood Borne and Sexually Transmitted Infections at University College, London, UK; 3 Bristol Medical School, Population Health Sciences, University of Bristol, Bristol, UK; 4 The NIHR HPRU in Behavioural Science and Evaluation at the University of Bristol, Bristol, UK; 5 UK Field Epidemiology Training Programme, UK Health Security Agency, Leeds, UK; 6 Virology, Microbiology Department, Leeds Teaching Hospitals Trust, Leeds, UK; 7 Department of Infection, Guy’s and St Thomas’ NHS Foundation Trust, London, UK; 8 Emergency Department, Guy’s and St Thomas’ NHS Foundation Trust, London, UK; 9 Public Health-Medical Department, Gilead Sciences Ltd UK and Ireland, London, UK

**Keywords:** Hepatitis B, Hepatitis C, Emergency department, Economic evaluation, Budget impact

## Abstract

**Background:**

Numerous studies have shown the effectiveness of testing for hepatitis B (HBV) and hepatitis C (HCV) in emergency departments (ED), due to the elevated prevalence amongst attendees. The aim of this study was to conduct a cost-effectiveness analysis of universal opt-out HBV and HCV testing in EDs based on 2 long-term studies of the real-world effectiveness of testing in 2 large ED’s in the UK.

**Methods:**

A Markov model was used to evaluate ED-based HBV and HCV testing versus no ED testing, in addition to current testing practice. The two EDs had a HBV HBsAg prevalence of 0.5–0.9% and an HCV RNA prevalence of 0.9–1.0%. The analysis was performed from a UK health service perspective, over a lifetime time horizon. Costs are reported in British pounds (GBP), and outcomes as quality adjusted life years (QALYs), with both discounted at 3.5% per year. Incremental cost-effectiveness ratios (ICER) are calculated as costs per QALY gained. A willingness-to-pay threshold of £20,000/QALY was used. The cost-effectiveness was estimated for both infections, in both ED’s.

**Results:**

HBV and HCV testing were highly cost-effective in both settings, with ICERs ranging from £7,177 to £12,387 per QALY gained. In probabilistic analyses, HBV testing was 89–94% likely to be cost-effective at the threshold, while HCV testing was 94–100% likely to be cost-effective, across both settings. In deterministic sensitivity analyses, testing remained cost-effective in both locations at ≥ 0.25% HBsAg prevalence, and ≥ 0.49% HCV RNA prevalence. This is much lower than the prevalence observed in the two EDs included in this study.

**Conclusions:**

HBV and HCV testing in urban EDs is highly cost-effective in the UK, and can be cost-effective at relatively low prevalence. These results should be reflected in UK and European hepatitis testing guidelines.

**Supplementary Information:**

The online version contains supplementary material available at 10.1186/s12962-022-00388-7.

## Introduction

Hepatitis B virus (HBV) and hepatitis C virus (HCV) can both result in chronic infections that lead to liver disease. If undiagnosed and untreated a significant number will progress to cirrhosis and hepatocellular carcinoma. The NHS has adopted the World Health Organisation (WHO) viral hepatitis elimination targets, which includes diagnosis of 90% of those living with the virus, and 80% of those eligible receiving treatment by 2030 [1]. In order to achieve these targets, hepatitis testing will need to be increased. In the UK, there were an estimated 118,000 people living with HCV as of 2019, with approximately two-thirds undiagnosed [2]. The number of people living with HBV in the UK is unknown, but estimates have ranged from 180,000 to 440,000 [3, 4].

Recent evidence has highlighted that the prevalence of HBV and HCV amongst emergency department (ED) attendees is considerably higher than that of the general population in the UK, with HBV HBsAg prevalence ranging from 0.5 to 3%, and HCV RNA prevalence ranging from 0.7 to 2.7% [5, 6]. Several studies have evaluated opt-out hepatitis testing for those attending EDs and receiving a blood test. Those who test positive are then contacted and linked to care if they are not engaged in care already. Testing studies have ranged from pilot studies, performing tests over one week, to longer studies in which testing was performed over 12 months [7–11]. The uptake of testing tended to be lower in studies over a longer duration (approximately 25% uptake amongst those eligible) [9–11].

A previous economic evaluation of hepatitis B and C testing in the ED has been performed using testing data from 2 studies performed in London [12]. The results of this evaluation found that ED testing for HBV and HCV were highly likely to be cost-effective at HBsAg and HCV RNA prevalence rates of 0.5% and above. However, there were several limitations with this analysis. Firstly, ED-based testing was either short in duration or had a low uptake amongst those eligible (56% uptake over 6 weeks, and 25% uptake over 9 months), and may not be reflective of testing and linkage to care rates when implemented at full scale [8, 9]. Second, the rates of testing and linkage to care were estimated from London EDs only, limiting the generalisability to other areas of the UK. Finally, the analysis did not consider the potential budget impact of an ED testing intervention, which will be of interest to those commissioning ED testing services.

The aim of this analysis was to update the previous cost-effectiveness analysis of ED-based universal opt-out HBV and HCV testing using long-term testing and linkage to care data from two urban settings in England, and to perform a budget impact assessment of implementation.

## Methods

### Model analyses and decision problem

The analysis evaluated the cost-effectiveness of ED testing in two locations; Leeds Teaching Hospitals NHS Trust (LTHT), and Guys and St. Thomas’ NHS Foundation Trust (GSTT) in London [13, 14]. In both EDs, the electronic patient record systems were modified to include a HBV and HCV test for adults, unless they opt-out. In LTHT, 25% of attendees received a test for urea and electrolytes (U and E) as part of their routine care and were therefore eligible for hepatitis testing. In GSTT, 46% of all ED attendees received a blood test as part of their routine care and were eligible for hepatitis testing. Of those eligible, the uptake of hepatitis testing was 57% and 75% in LTHT and GSTT respectively. A decision model was developed to evaluate the cost-effectiveness and budget impact of opt-out HBV and HCV testing for those attending the EDs. The comparator was no hepatitis testing in the ED, meaning those infected and undiagnosed remained so, at least until they receive testing in another setting in the future. The model evaluated the cost-effectiveness of HBV and HCV testing as 2 separate decisions, each compared to no hepatitis testing in the ED. This is reasonable as there were no shared costs between the tests, and so testing for one virus may be cost-effective whilst testing for the other may not be.

The cost-effectiveness analysis was performed from a UK National Health Service (NHS) perspective. Costs are presented in British pounds (GBP) in 2020 prices, with costs inflated using the NHS inflation index where necessary [15]. Health outcomes are presented as quality-adjusted life years (QALYs). The model was run over a lifetime time horizon, with an annual cycle length. Costs and outcomes are discounted at 3.5%, as per National Institute for Health and Care Excellence (NICE) guidelines [16]. The model estimates the incremental cost-effectiveness ratio (ICER) by dividing the incremental costs by the incremental QALYs of providing hepatitis testing compared to no testing. A simple budget impact analysis was also performed from a UK NHS perspective. This analysis primarily used the decision tree of the cost-effectiveness model to evaluate the costs associated with the intervention, which were assumed to be over a one-year period.

### Model structure

The cost-effectiveness model used in this analysis is an adaptation of a previous decision tree and 2 Markov models used to evaluate hepatitis testing in EDs [12]. The Markov model structures are shown in the Additional file 1.

The decision tree captures the components of ED testing, including the prevalence of infection as indicated by the diagnostic test (HBsAg + , HCV RNA + , or uninfected), the proportion of patients requiring linkage to care (i.e. new diagnoses or those previously disengaged from care), and actual linkage to care (including the proportion attending clinic and proportion receiving treatment, if indicated). The decision tree informs the starting health state in the appropriate Markov model.

For HCV, people in early disease states (up to and including compensated cirrhosis) have the opportunity to receive treatment and achieve a sustained virological response (SVR). For HBV, those diagnosed can engage in care, which assumes they receive and adhere to treatment if indicated based on their clinical status, or can disengage with care, in which case they are assumed to receive no treatment. Those with HBV or HCV follow the progressive nature of these diseases, captured through Markov health states.

### Model population

The mean age of individuals entering the model with HBV was 41.1 and 48.6, and with HCV was 42.3 and 47.1, as derived from LTHT and GSTT hospitals, respectively (Table 1).

**Table 1:** Key intervention and clinical parameters

Base case probabilities	Mean value (LTHT, Leeds)	Distribution	Mean Value (GSTT, London)	Distribution	Source
HBV Parameters					
Age of HBV cases	41.1	N/A	48.6^a^	N/A	LTHT [13]/GSTT [14]
Prevalence (HBsAg)	0.5%	Beta (α = 73, β = 15,980)	0.9%	Beta (α = 235, β = 27,411)	LTHT/GSTT
Proportion of diagnoses requiring linkage to care	53.4%	Beta (α = 39, β = 34)	57.4%	Beta (α = 135, β = 100)	LTHT/GSTT
Proportion attending referral	69.2%	Beta (α = 27, β = 12)	71.1%	Beta (α = 96, β = 39)	LTHT/GSTT
Proportion accepting treatment, post-referral (if indicated)	86.6%	Beta (α = 13, β = 2)	86.6%	Beta (α = 13, β = 2)	Parry [9]
Proportion HBeAg +	8.3%	Beta (α = 5, β = 55)	8.3%	Beta (α = 5, β = 55)	LTHT [13]
Proportion inactive disease (HBeAg + seroconverted or HBeAg- inactive disease)	80%	Beta (α = 80, β = 20)	80%	Beta (α = 80, β = 20)	PHE
Proportion cirrhotic	12%	Beta (α = 3, β = 22)	12%	Beta (α = 3, β = 22)	Parry
HCV Parameters					
Age of HCV cases	42.3	N/A	47.1^a^	N/A	LTHT/GSTT
Prevalence (HCV RNA + or Ag +)	1.0%	Beta (α = 156, β = 15,897)	0.9%	Beta (α = 261, β = 27,396)	LTHT/GSTT
Proportion of Ab + testing RNA + /Ag + upon reflex test	45.6%	Beta (α = 156, β = 186)	49.9%	Beta (α = 261, β = 262)	LTHT/GSTT
Proportion of diagnoses requiring linkage to care	94.9%	Beta (α = 148, β = 8)	86.5%^b^	Beta (α = 217, β = 34)^b^	LTHT/GSTT
Proportion attending referral	51.4%	Beta (α = 76, β = 72)	23.5%	Beta (α = 51, β = 166)	LTHT/GSTT
Proportion receiving treatment, post-referral	53.9%	Beta (α = 41, β = 35)	51.0%	Beta (α = 26, β = 25)	LTHT/GSTT
Proportion F0	57.5%	Dirichlet (42, 12, 2, 5, 12)	22.7%	Dirichlet (**10**,10,10,7,7)^c^	LTHT/Parry
Proportion F1	16.4%	Dirichlet (42, 12, 2, 5, 12)	22.7%	Dirichlet (10,**10**,10,7,7)^c^	LTHT/Parry
Proportion F2	2.7%	Dirichlet (42, 12, 2, 5, 12)	22.7%	Dirichlet (10,10,**10**,7,7)^c^	LTHT/Parry
Proportion F3	6.8%	Dirichlet (42, 12, 2, 5, 12)	15.9%	Dirichlet (10,10,10,**7**,7)^c^	LTHT/Parry
Proportion cirrhotic (F4)	16.4%	Dirichlet (42, 12, 2, 5, 12)	15.9%	Dirichlet (10,10,10,7,**7**)^c^	LTHT/Parry
Proportion current PWID	61.3%	Beta (α = 84, β = 53)	61.3%	Beta (α = 84, β = 53)	LTHT

Bold values indicate the element of the Dirichlet distribution (i.e. first value represents F0, second value F1 etc.)

^a^The mean age for individuals diagnosed in GSTT was estimated from the proportion of patients in each age band, using a midpoint to calculate the mean age

^b^Patients who were uncontacted were assumed to require linkage to care

^c^Sample size of 44, Dirichlet (10,10,10,7,7)

Since injecting drug use is a major risk factor for HCV, the HCV model differentiated between people who inject drugs (PWID) and other individuals in the model. Data on the proportion of current PWID was available from LTHT (61%), and in the absence of data for GSTT, this proportion was also used for GSTT hospital. A sensitivity analysis considered data from another London based testing study which reported a slightly lower proportion of current or ex-PWID (54.5%), albeit from a sample of just 11 patients [9]. In the base case model, the disease progression, background risk of mortality, reinfection rate, and the background probability of receiving testing were all different for current PWID (see Additional file 1: Table S1 and S4 for parameter details).

### Prevalence and linkage to care

Amongst those receiving ED testing, 0.5% in LTHT and 0.9% in GSTT were HBV HBsAg positive. The HCV RNA prevalence was 1.0% in LTHT and 0.9% in GSTT (in GSTT all HCV antigen positive confirmatory tests were HCV RNA positive). The model assumes those testing HCV antigen positive are RNA positive. The proportion of HCV antibody positive tests which tested HCV RNA or antigen positive was similar in both locations (46–50%).

The linkage to care parameters were also derived from each ED. Amongst those testing positive, only those undiagnosed or previously diagnosed but not currently engaged in care required linkage to care. This was 53–57% for HBV, and 87–95% for HCV. If a patient remained uncontacted and did not have evidence of being a known diagnosis and already being engaged in care, the model assumed that they required linkage to care.

Patients requiring linkage to care in both EDs were contacted by various means, including phone calls, text messages, and letters to their home and registered GP. Full details are available in the original testing publications [13, 14]. Specialist outreach nurses or teams working with the homeless were also informed of those requiring linkage to care. The model captures the proportion that were engaged in care, defined as those who returned for at least one hospital appointment. For HBV, this was 69.2% in LTHT, and 71.1% in GSTT. For HCV, it was 51.4% in LTHT and 23.5% in GSTT.

For HBV patients, there was no data on the proportion that were offered treatment, or who accepted treatment. For both settings, we assumed 86.6% would accept treatment if offered, based on a separate ED testing study [9]. Treatment was only provided when clinically indicated (see treatment section below). For HCV, a proportion of those engaged would receive direct acting antiviral (DAA) treatment, and this was available from both settings. In LTHT, 53.9% of those engaged received treatment, with 51% receiving treatment in GSTT.

In GSTT, patient information was shared with local hospital homeless services, and the Find and Treat team (UCLH NHS Trust), a pan-London community inclusion health outreach team. This was successful in linking those testing positive to treatment, and is included within the above linkage to care data. A similar approach was taken in LTHT, with specialist nurse teams helping to find those with no fixed abode in GP practices and shelters, and in drug and alcohol services.

The severity of fibrosis was only available for those with HCV from LTHT. For GSTT, the fibrosis levels were derived from another ED testing study in London, although the values from LTHT were used in a sensitivity analysis [9]. The proportion of cirrhotic HBV patients was also derived from this study, since this data was not available in LTHT or GSTT.

### Treatment parameters

Treatment for HBV followed NICE guidelines, with HBeAg + patients and those with HBeAg- active disease receiving pegylated interferon alpha-2a (PegIFN) for one year, followed by tenofovir disoproxil fumarate (TDF) if treatment continued [17]. Treatment sought to achieve HBeAg seroconversion or inactive disease. Full details of HBV treatment are available in the Additional file 1. Since many of those receiving HBV treatment will require long-term treatment, we included an annual probability of treatment disengagement (3.3% per year), to avoid overestimating the benefit associated with treatment.

All HCV patients from F0 to compensated cirrhosis health states accepting treatment were assumed to receive a pan-genotypic DAA. An estimated 93% of non-cirrhotic and 91% of cirrhotic patients achieve an SVR, based on UK outcome data [18]. We assumed that those not achieving an SVR would be re-treated once, with SVR rates of 95.3% and 81% for non-cirrhotics and cirrhotics, respectively [19].

### Transition probabilities

The transition probabilities for the HBV model were derived from a UK Health Technology Assessment, and differed by HBeAg status [20]. Those on treatment were more likely to achieve inactive disease or HBeAg/HBsAg seroconversion which slowed disease progression, compared to those not receiving treatment. For early HCV states (F0 to compensated cirrhosis), transition probabilities were derived from a meta-regression of HCV progression rates, which differed for PWID and non-PWID [21]. For more advanced disease states (compensated cirrhosis progression onwards), transition probabilities were derived from a UK study [22]. For those achieving SVR, disease progression either halted (F0-F3 health states) or dramatically reduced (compensated cirrhosis state) [23, 24]. For HCV PWID, a standardised mortality ratio of 7.8 was also applied to the probability of death, which was applied for 11 years, the estimated duration of injecting [25, 26]. All transition probability values are available in the Additional file 1. Both models also include the risk of all-cause mortality in each cycle, based on UK life tables [27].

### Background probability of testing

The background probability of testing for HBV and non-PWIDs with HCV was estimated by dividing the number of tests recorded in the PHE sentinel surveillance statistics of blood-borne virus testing in England (after adjusted for database coverage), by estimated adult population in England [28, 29]. The background probability of HBV testing was estimated to be 2.5% per year, from all HBV tests, except paediatric tests. For HBV, we adjusted the frequency in which those infected would be tested in the background rate of testing, to ensure that the proportion of positive tests was equal to the national average of 1.0% [28].

For non-PWID HCV diagnoses, the background probability of testing was estimated to be 2.1%, based on all HCV tests except paediatric tests and tests from setting which were likely to test PWID (drug dependency services, prisons, and pharmacies). For PWIDs, an estimated 26.8% received testing each year, from the PHE Unlinked Anonymous Monitoring survey of PWID [30]. We did not adjust the testing rates for HCV, as the background rate of testing was already elevated amongst PWID.

### Costs

The costs associated with the intervention, including test costs, treatment related costs, and costs of healthcare appoints are provided in Table 2.

**Table 2:** Intervention and treatment costs

Costs (per year, except where noted)	Cost	Cost year	Distribution	Source
LTHT, Leeds				
HBsAg test	£2.26	2019/20	N/A	LTHT [13]
HBsAg confirmation test	£13.36	2019/20	N/A	LTHT
HCV antibody test (initial)	£4.19	2019/20	N/A	LTHT
HCV antibody confirmation test	£9.58	2019/20	N/A	LTHT
HCV RNA test	£17.72	2019/20	N/A	LTHT
Nurse cost to contact positive case	£55.41	2019/20	N/A	Curtis [15]
GSTT, London				
HBsAg test & confirmation	£5.79	2019/20	N/A	GSTT [14]
HCV antibody test + HCV antigen confirmation test	£6.67	2019/20	N/A	GSTT
HCV RNA test	£73.87^a^	2015/16	N/A	Bradshaw [10]
Nurse cost to contact positive case	£40.10	2019/20	N/A	Curtis [15]
Find and Treat—Engagement	£75.18^a^	2017/18	Uniform(£60.14, £90.21)	Ward [32]
Find and Treat—Peer support for hospital visit	£126.32^a^	2017/18	Uniform(£101.06, £151.59)	Ward [32]
Both locations				
PegIFN (Annual)	£3672	2019/20	N/A	BNF [34]
TDF (Annual)	£366	2019/20	N/A	BNF [34]
TDF + Emtricitabine (Annual)	£1299	2019/20	N/A	BNF [34]
DAA treatment	£10,000	2019/20	N/A	Hurley [35]
DAA re-treatment	£15,000	2019/20	N/A	Hurley [35]
Cost of background test appointment	£33.19	2019/20	Uniform(£16.60, £49.79)	Curtis [15]
Pre-treatment evaluation (initial)	£207.86	2019/20	Uniform(£168.80, £253.20)	NHS reference costs 2019/20 [33]
Pre-treatment evaluation (follow-up)	£164.75	2019/20	Uniform(£156, £234)	NHS reference costs 2019/20
DAA treatment monitoring	£823.75	2019/20	Uniform(£780, £1170)	NHS reference costs 2019/20

^a^Costs presented have been inflated to 2019/20, using NHS cost inflation index (2015/16 = 1.08, 2017/18 = 1.046)

#### Test costs

The test costs were derived from each ED separately. In LHTH, costs were estimated using the base test cost, plus the laboratory add-on costs and bio-medical scientist time. In GSTT, individual test costs were provided. HBV testing consisted of a HBsAg test, plus a confirmatory test for those who test positive. For LTHT, these costs were incurred separately (£2.26 and £13.36 respectively), whilst in GSTT, a single cost was applied for each test (£5.79), whether a confirmatory test was required or not. The HCV testing approach differed in each setting. In LTHT, a HCV antibody test was performed (£4.19), with a confirmatory antibody test performed for any initial positive test (£9.58). Following two positive antibody tests, patients received an RNA test (£17.72). In GSTT, HCV testing consisted of an HCV antibody test, followed by a confirmatory HCV antigen test for those antibody positive, with a total cost of £6.67 (whether confirmatory testing occurred or not). For those testing antigen positive, an RNA test was performed, although the source of this cost was another ED testing study from London (£73.87) [10]. The costs of additional tests for viral markers or genotype were assumed to be included in the cost of the hospital visit, for those linked to care.

#### Contacting patients and healthcare costs

The model assumed that all patients testing positive were contacted, whether they required linkage to care or not. The costs of contacting patients from the ED was estimated from the salary costs of part-time nurses in both locations (band 6, 0.5 full-time equivalent for LTHT, band 7, 0.4 full-time equivalent for GSTT). Salaries were derived from UK personal social services, and divided by the number of positive tests to contact in each setting, giving an average cost of £55.41 per positive case in LTHT, and £40.10 in GSTT [15]. The costs were assumed equal for contacting HBV and HCV cases, although for HCV additional costs for outreach activities are applied. A sensitivity analysis considered a higher cost to contact each positive case, with a full-time band 6 nurse assumed to be contacting cases, with an average cost of £110.83 in LTHT, and £81.66 in GSTT, per positive case.

In addition, for HCV we included the cost of an inclusion health team, who were responsible for outreach services to link patients to care, based on collaboration between GSTT and UCL Find and Treat, with costs derived from a previous economic evaluation of their service [31, 32]. The average costs of engaging RNA positive patients was £75.18 per person, and peer support for engagement for each hospital visit was £126.32 per person. This was applied to 31.3% of those requiring linkage to care (31/99), and 37.3% of those requiring a hospital visit (19/51), based on data from GSTT. To be conservative, the same costs and proportions were applied to LTHT, since nurses had close contact with specialist GPs for the homeless, and close links to drug and alcohol centres, but no data for proportions were recorded as these links were informal and existed previously.

For HBV and HCV patients returning and engaged in care, the model assumes an initial healthcare visit (£207.86) derived from NHS reference costs [33]. For HBV, those who are fully engaged (and would receive treatment if indicated) incurred a second visit cost (£164.75), whether they did receive treatment or not, based on their health status [33]. For HCV, those returning for DAA treatment also incurred a second visit cost (£164.75).

#### Treatment costs

HBV treatment costs were derived from the British National Formulary (BNF) [34]. For HCV, the NHS has negotiated a confidential price reduction on the costs of DAA treatment, expected to be approximately £5000 per successful treatment [35]. To remain conservative, we assumed a cost of £10,000 per DAA treatment, and £15,000 for re-treatment, with the cost incurred only upon SVR, as per NHS policy [36]. We explored lower DAA costs in sensitivity analyses. DAA treatment monitoring costs were assumed to consist of five visits for HCV patients (£823.75) [33]. HBV treatment monitoring was assumed to be included in the specific health state costs.

#### Health state costs

The health state costs for HBV and HCV were derived from two UK HTA reports and other literature sources (Additional file 1: Table S5) [20, 22, 37]. The health state costs were only incurred for those patients diagnosed. Health state costs were lower for those achieving seroconversion or inactive disease (HBV) or achieving SVR (HCV).

### Utility

Utility values were assigned to each health state to estimate the health-related quality of life. For early HBV health states (pre-cirrhotic), utilities were derived from a study of over 400 HBV patients [38]. For early HCV health states, utilities were derived from a large meta-analysis of studies in HCV patients, with utility estimated using the EQ-5D-3L [39]. The same source was used for later health states (compensated cirrhosis onwards), and the utility values for each health state were assumed the same for HBV and HCV patients [39].

### Cost-effectiveness analyses

One-way deterministic sensitivity analyses were performed on parameters of interest, by individually changing parameter values and observing the impact upon the ICER. A probabilistic sensitivity analysis (PSA) was performed to capture the parameter uncertainty in the model. Distributions were assigned to appropriate model parameters, with each sampled simultaneously across 10,000 Monte Carlo simulations. The parameters included, and distributions used, are provided in Table 1 and Table 2, and Additional file 1: Tables S1–6.

A probabilistic threshold analysis was also performed, to consider the minimum prevalence at which HBV and HCV testing would remain 90% cost-effective. This involved running the same PSA as described above, with prevalence values increasing by 0.05% increments. This has been described as a two-level Monte Carlo approach [40]. This was performed for both settings individually, and when combining results for the two settings.

### Budget impact analysis

A budget impact analysis was performed to estimate the costs associated with HBV and HCV testing and linkage to care. The analysis assumes testing is performed over a one year period (at the same testing rate as the 2 studies, since both studies were performed for less than a year) [13, 14]. For both HBV and HCV, the budget impact estimated the costs of the intervention, including all tests, contacting positive patients, and healthcare visits for those engaging with care (Table 2). The budget impact analysis only focused on the ED-based costs of the intervention, including testing and linking patients into care, and therefore did not consider treatment costs.

## Results

### Base case analysis

The base case results show that testing for HBV and HCV was cost-effective in both settings (Table 3). For HBV testing, testing in LTHT resulted in an incremental cost of £12.12 and 0.00125 QALYs per person tested, with an ICER of £9728 per QALY gained. In GSTT, it resulted in an incremental cost of £31.04 and 0.00316 QALYs, with an ICER of £9833 per QALY gained. For HCV testing, testing in LTHT resulted in an incremental cost of £13.18 and 0.00184 QALYs per person tested, with an ICER of £7177 per QALY gained. In GSTT, testing resulted in an incremental cost of £10.13 and 0.0008 QALYs, with an ICER of £12,387 per QALY gained.

**Table 3:** Base case cost-effectiveness results for HBV and HCV testing, per person receiving testing, in LTHT and GSTT

Location	Testing strategy	Mean costs	Mean QALYs	Incremental costs	Incremental QALYs	ICER per QALY gained
HBV Testing						
LTHT, Leeds	No ED testing	£60.29	16.531			
	ED Testing	£72.41	16.532	12.12	0.00125	£9728
GSTT, London	No ED testing	£97.55	14.670			
	ED Testing	£128.58	14.673	31.04	0.00316	£9833
HCV Testing						
LTHT, Leeds	No ED testing	£115.74	16.233			
	ED Testing	£128.93	16.235	13.18	0.00184	£7177
GSTT, London	No ED testing	£120.12	14.917			
	ED Testing	£130.25	14.918	10.13	0.00082	£12,387

### Deterministic sensitivity analyses

**Fig. 1:**
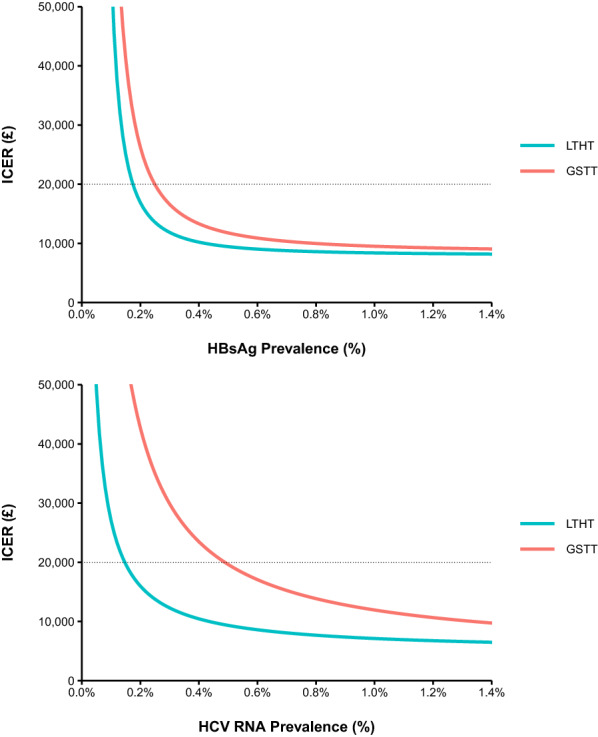
Deterministic Sensitivity Analysis considering cost-effectiveness of HBV and HCV testing across a range of HBsAg prevalence and HCV RNA prevalence, in LTHT Leeds and GSTT London

In the deterministic sensitivity analysis, the minimum HBsAg prevalence at which HBV testing was cost-effective was 0.18% in LTHT and 0.25% in GSTT (Fig. 1). HCV testing was cost-effective when HCV RNA prevalence was 0.15% or higher in LTHT, and 0.49% or higher in GSTT.

For both HBV and HCV testing, the proportion of patients attending their referral following a positive test had the biggest impact upon the ICER. This was the only scenario which resulted in an ICER above the £20,000/QALY threshold (when assuming only 11.8% of HCV positive patients attended their referral in GSTT). Older age, and lower fibrosis scores (in HCV patients) also reduced the cost-effectiveness. Full results are available in the Additional file 1.

### Probabilistic analyses

**Fig. 2:**
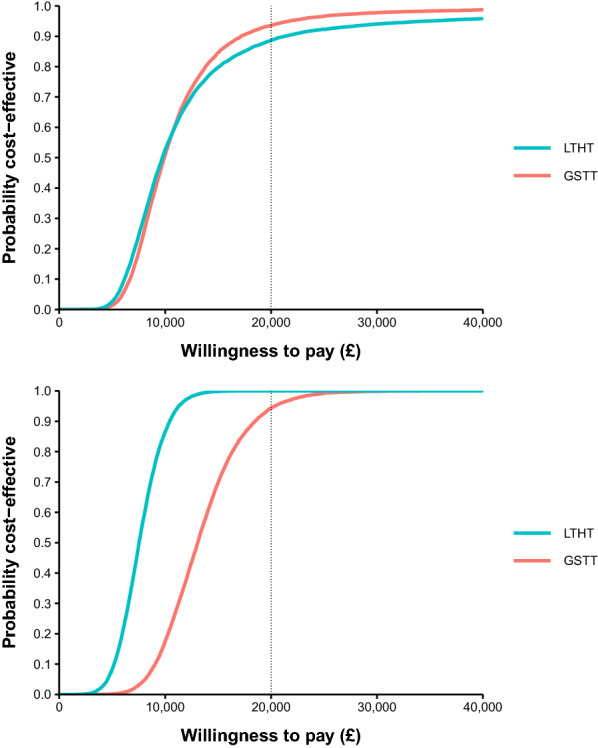
Cost-effectiveness acceptability curves (CEAC) for HBV testing (top) and HCV testing (bottom) in LTHT Leeds, and GSTT London

In the PSA, HBV and HCV testing was highly likely to be cost-effective in both settings. HBV testing was 88.7% and 93.6% likely to be cost-effective in LTHT and GSTT, respectively, at the £20,000 per QALY threshold. HCV testing was 100% and 94.3% likely to be cost-effective in LTHT and GSTT, respectively (Fig. 2).

In the probabilistic threshold analysis, the minimum prevalence at which testing remained 90% likely to be cost-effective across both settings was 0.6% HBsAg prevalence, and 0.75% HCV-RNA prevalence, at a £20,000 per QALY threshold.

### Budget impact analysis

Table 4 shows the annual budget impact for each setting, with an estimated 21,404 HBV and HCV tests in LTHT, and 30,171 HBV and HCV tests in GSTT. For HBV testing, the total budget impact in LTHT was £67,690 for the testing, contacting and linkage to care, resulting in approximately 36 patients engaged in care over one year. In GSTT, the total budget impact was £221,710, with approximately 104 patients engaged. The budget impact of HCV testing was £152,728 in LTHT, with an estimated 54 patients treated. In GSTT, the budget impact of HCV testing was £280,208, with 29 patients treated. The test costs were the largest cost component across both testing strategies and both settings.

**Table 4:** Budget impact analysis of annual HBV and HCV testing

Budget impact item	LTHT, Leeds^a^	GSTT, London^a^
HBV Testing		
Testing costs	£49,673	£174,690
Cost of contacting positive cases	£5393	£10,284
Appointment costs for those engaged	£12,623	£36,736
HBV Testing total	£67,690	£221,710
HCV Testing		
Testing costs	£102,132	£243,389
Cost of contacting positive cases	£11,525	£11,417
Appointment costs for those engaged	£30,069	£16,884
Additional outreach costs (Find and Treat)	£9002	£8517
HCV Testing total	£152,728	£280,208

^a^Annual testing assumes 21,404 HBV and HCV tests per year in LTHT, and 30,171 HBV and HCV tests per year in GSTT

When assuming a full-time band 6 nurse was needed to contact patients, the budget impact only increased marginally, as this represents a small proportion of the overall cost (see Additional file 1 for a breakdown of the costs).

## Discussion

### Main findings and considerations for implementation

Our analysis used testing data from the two of the largest ED testing studies in the UK to date, with 16,000 and 27,600 tests performed in each setting, and linkage to care outcomes reflective of a real world implementation of hepatitis testing in the ED. Our results showed that HBV and HCV testing in the ED was highly likely to be cost-effective in both areas, with little uncertainty in the cost-effectiveness results across a range of sensitivity analyses. These results provide further economic evidence to support the implementation of ED testing in areas with a modest prevalence of HBV and HCV.

When considering implementing hepatitis testing across UK EDs, the key factors to consider are the linkage to care achieved by the testing intervention, the prevalence of infection amongst attendees, and the cost of the tests used. Our threshold analyses show that testing can be cost-effective at lower prevalence than observed in the 2 EDs. The minimum prevalence for testing to be cost-effective in both settings was 0.25% HBsAg prevalence and 0.49% HCV RNA prevalence (Fig. 1). These are slightly higher than our estimates from a previous study, and likely more reflective of the real-world implementation of ED testing for HBV and HCV [12]. That said, testing for HCV in Leeds was cost-effective at a much lower HCV RNA (0.15%), likely due to the lower test costs and higher linkage to care.

Prior to the introduction of a testing intervention, it is important that the pathways for engaging those who test positive are in place, since the linkage to care is critical to the cost-effectiveness of the intervention, as demonstrated in our sensitivity analyses. Both EDs engaged with local outreach teams, who were influential in linking patients to care. Moreover, the costs of staff to link patients to care were relatively small cost components of the intervention. If additional resources for staff can improve the proportion of patients linked to care then this is likely to be a worthwhile investment. The main cost of the intervention were the diagnostic test costs, so if lower prices can be negotiated based on the high volume of tests performed, it would increase the affordability and cost-effectiveness of testing (Table 4).

### Comparison between EDs

There were a number of differences between the two EDs which influenced the cost-effectiveness results. For HBV, the proportion of patients linked to care was similar, although the prevalence was considerably higher in GSTT compared to LTHT. This is unsurprising, given previous UK reports have shown London has the highest incidence of HBV across English regions [41]. Despite the differences in the parameters, the ICERs for HBV strategies were similar across locations, likely due to the lower test costs in Leeds, and the slightly younger average age.

The HCV RNA prevalence was similar in both settings, but the proportion attending their referral was higher in LTHT compared to GSTT. This may be partly explained by better integration of services for people who are homeless or with addiction needs in smaller cities such as Leeds. In comparison, there are 18 acute NHS trusts in London, and many different services for people who are homeless or use drugs, creating challenges around communication and collaboration [42]. It should also be noted that LTHT had a slightly higher proportion of a full-time nurse to contact positive cases (0.5 for LTHT vs. 0.4 for GSTT), and a lower number of people to contact per month. The higher linkage to care rates in Leeds, alongside the lower test costs, and slightly lower mean age, resulted in a considerably lower ICER compared to GSTT.

### Comparisons with other research

Testing for HIV in the ED is currently recommended by NICE guidelines at a prevalence of 0.2% or above, but no such recommendations exist in NICE hepatitis B and C testing guidelines [43, 44]. Hepatitis B and C testing is already recommended by the European Centre for Disease Prevention and Control (ECDC) when the prevalence exceeds 2%, which is considerably higher than the prevalence threshold at which testing is likely to be cost-effective in our analysis [45].

In addition to the studies used in our analysis, evidence around the effectiveness of ED testing has been demonstrated across several studies in the UK, including pilot studies, national ‘testing weeks’, and long term testing studies [8–11]. Qualitative analyses in the UK also found ED testing to be acceptable, and a valuable practice for patients and staff [46].

Various approaches to ED testing for hepatitis have also been reported in many high income countries across Europe and North America, although many are targeted towards those at risk (drug users, or those in birth cohorts), rather than universal opt-out testing [47–56]. Most of these studies focused on HCV and HIV testing, with few testing for HBV.

Our previous cost-effectiveness analysis showed that HBV and HCV testing is likely to be cost-effective in EDs in the UK, even at a relatively modest prevalence. However, the initial analysis had a number of limitations, which have been improved upon. We have used data from two long-term testing studies in two different cities in the UK, with a higher uptake of testing compared to the previous studies. This is likely to be reflective of the impact of real-world universal opt-out ED testing using electronic patient record based testing [13, 14]. We also provide a budget impact analysis for decision makers who are considering the cost of implementing ED based testing.

Only one other economic evaluation was identified, which evaluated ED based testing for HCV performed in Canada [51]. Testing for all ED attendees was highly likely to be cost-effective. The ICER’s reported are higher than those in our analysis, but testing remained cost-effective at a 1% HCV RNA prevalence. The higher ICERs are likely to be partially explained by the much higher costs of antibody screening tests (CA$24 versus £4.19–£6.67 in our study), and DAA treatment costs (CA$60,000 versus £10,000).

### Limitations

This analysis builds on a previous economic analysis performed in the UK, but several limitations remain.

One limitation is the uncertainty around the extent to which ED testing is likely to identify people who could be tested elsewhere. If those being identified in the ED are likely to be tested in other services shortly after, then the benefit of testing may be lower than estimated in this analysis. This is particularly true for HCV testing, which has increased over recent years, particularly for people who currently, or have recently, injected drugs. However, given a considerable number of diagnoses were new or previously not engaged in care, this suggests that there is still a need for testing to identify and engage these patients. Sensitivity analyses considering higher and lower testing rates in non-PWIDs had little impact on the ICER. We did not evaluate the impact of higher background rate of testing among PWID since this was based on high quality UK data, and already high (26.8% per year) [30]. Although PWID had a higher background rate of testing (reducing the ICER), the model did not consider the prevention benefit of reduced onward transmission associated with earlier diagnosis and treatment of HCV amongst PWID, despite including a reinfection rate for PWIDs achieving SVR. This is likely to overestimate the ICER for HCV testing.

The model assumes that the linkage to care for those testing in other settings was equal to the linkage to care achieved in the ED. We did not explicitly consider other settings in which testing could occur, or whether the linkage to care may be higher in these settings. A more sophisticated modelling approach could overcome this, but it would need to track individuals through each healthcare setting or service that they attend. The model would need data on the probability of testing and linkage to care, and the intervention costs, for every possible setting, which would be a very complex analysis to undertake.

The nurse costs for contacting patients were derived from each ED, but may underestimate the costs of staff time associated with testing. A sensitivity analysis using higher staff costs for contacting patients, assuming a full-time band 6 nurse, had little impact upon the cost-effectiveness or budget impact results.

Finally, our analysis did not consider differences in the designs or implementations of testing within the EDs. The approaches taken to link patients into care evolved over the course of the intervention in both EDs. Future studies should consider the best methods to engage with patients, including the type of staff and resources required to contact patients, and the partnerships with other services (e.g. outreach services) needed to successfully link patients to care. These will also influence the cost-effectiveness of testing too.

## Conclusion

Universal opt-out HBV and HCV testing in the ED was highly cost-effective in both settings. Testing was cost-effective in both settings if the HBsAg prevalence was 0.25% or above, and the HCV RNA prevalence is 0.49% or above. NICE hepatitis testing guidelines should be updated to recommend testing in areas with elevated prevalence, similar to the testing guidelines for HIV [43]. Testing may also be cost-effective in other areas of Europe, and was highly cost-effective at a much lower prevalence than the 2% prevalence thresholds recommended by the ECDC testing guidelines. Further research should focus on the best ways for ED testing interventions to contact and link patients to care, since this had a large impact on the cost-effectiveness.

## Supplementary Information

**Additional file 1: Fig. S1.** HBV Markov model structure. **Fig. S2.** HCV Markov model structure. **Fig. S3.** Cost-effectiveness of HCV testing in LTHT Leeds, and GSTT London, across a range of DAA treatment costs. **Table S1.** Other model probabilities. **Table S2.** Base case HBV transition probabilities for individuals entering the model with chronic HBV and HBeAg positive disease. **Table S3.** Base case HBV transition probabilities for individuals entering the model with chronic HBV and HBeAg negative disease. **Table S4.** HCV Transition probabilities. **Table S5.** Health state costs. **Table S6.** Base case utility values. **Table S7.** One-way deterministic sensitivity analyses of HBV testing, in LTHT Leeds and GSTT London. **Table S8.** One-way deterministic sensitivity analyses of HCV testing, in LTHT Leeds and GSTT London. **Table S9.** Minimum prevalence at which HBV and HCV testing in the ED are 90% likely to be cost-effective in probabilistic analyses, for each setting, and across settings. **Table S10.** Sensitivity analysis of budget impact of annual HBV and HCV testing assuming cost of contacting positive cases performed by full time band 6 nurse

## Data Availability

Data sharing is not applicable to this article as no datasets were generated or analysed during the current study. The data used for this model is publicly available.

## Abbreviations

HBV: Hepatitis B virus
HCV: Hepatitis C virus
ED: Emergency department
GBP: Great British pounds
QALY: Quality adjusted life years
ICER: Incremental cost-effectiveness ratios
WHO: World Health Organisation
LTHT: Leeds Teaching Hospitals NHS Trust
GSTT: Guys and St. Thomas’ NHS Foundation Trust
NHS: National Health Service
SVR: Sustained virological response
PWID: People who inject drugs
DAA: Direct acting antiviral
PegIFN: Pegylated interferon alpha-2a
TDF: Tenofovir disoproxil fumarate
BNF: British National formulary
PSA: Probabilistic sensitivity analysis
ECDC: European Centre for Disease Prevention and Control
