# Supplementary material for: Universal testing for hepatitis B and hepatitis C in the emergency department: a cost-effectiveness and budget impact analysis of two urban hospitals in the United Kingdom
Source: Cost Eff Resour Alloc. 2022 Nov 14;20:60. doi: 10.1186/s12962-022-00388-7 (PMC9664679; doi:10.1186/s12962-022-00388-7)
Supplement: Supplementary file 1 — Additional file 1: Fig. S1. HBV Markov model structure. Fig. S2. HCV Markov model structure. Fig. S3. Cost-effectiveness of HCV testing in LTHT Leeds, and GSTT London, across a range of DAA treatment costs. Table S1. Other model probabilities. Table S2. Base case HBV transition probabilities for individuals entering the model with chronic HBV and HBeAg positive disease. Table S3. Base case HBV transition probabilities for individuals entering the model with chronic HBV and HBeAg negative disease. Table S4. HCV Transition probabilities. Table S5. Health state costs. Table S6. Base case utility values. Table S7. One-way deterministic sensitivity analyses of HBV testing, in LTHT Leeds and GSTT London. Table S8. One-way deterministic sensitivity analyses of HCV testing, in LTHT Leeds and GSTT London. Table S9. Minimum prevalence at which HBV and HCV testing in the ED are 90% likely to be cost-effective in probabilistic analyses, for each setting, and across settings. Table S10. Sensitivity analysis of budget impact of annual HBV and HCV testing assuming cost of contacting positive cases performed by full time band 6 nurse [file 12962_2022_388_MOESM1_ESM.docx]

**Universal testing for hepatitis B and hepatitis C in the Emergency Department: A cost-effectiveness and budget impact analysis of two urban hospitals in the United Kingdom:**

**Appendix**

## Hepatitis B treatment

The transition probabilities for the natural history of HBV were largely based upon a HTA performed by Shepherd *et al*.[1] Treatment related transition probabilities were sourced from various clinical trials, specific to HBeAg status (details below). Since treatment can be for long durations, the model assumes that 3.33% of individuals per year would disengage with care, moving from ‘engaged in care’ or ‘on treatment’ health states to ‘not engaged in care’ health states. This was based on a study by Marcellin, capturing all individuals lost or excluded from the final analysis for any reason other than a clinical justification (such as a treatment switch due to tolerability or off treatment subsequent to seroconversion).[2]

### HBeAg positive HBV

Based on NICE treatment guidelines for chronic HBV, it was assumed that HBeAg positive patients identified with active disease were treated with Peginterferon Alfa-2a (PegIFNα) for 48 weeks (assumed one model cycle).[3] The model assumes 32% of individuals achieve e-antigen seroconversion after one year of treatment, whilst 3% achieve HBsAg seroconversion.[1, 4]

For those that do not achieve e-antigen seroconversion, patients were treated with tenofovir disoproxil fumarate (TDF) until e-antigen seroconversion was achieved.[5] For HBeAg positive patients, 13% were assumed to receive Emtricitabine alongside TDF.[6] The annual probability of those e-antigen seroconverting whilst treated with TDF was estimated to be 5.6%. This was derived from a long term study of TDF, with seroconversion calculated between weeks 48 and 240 and converted to an annual probability.[6] The annual probability of HBsAg seroconversion was estimated to be 1.8% on TDF.[6] The transition probabilities associated whilst on treatment (PegIFNα or TDF) were assumed to be the same as for individuals achieving e-antigen seroconversion, due to the relatively high virological control and decrease in HBV DNA whilst on treatment.[4, 6] This is supported by long term data in which 98% of individuals treated with TDF achieved a virological response at 8 years.[2] HBeAg positive individuals with no cirrhosis were assumed to cease treatment upon e-antigen seroconversion. For cirrhotic individuals, treatment with TDF was assumed to continue, even after e-antigen seroconversion.

### HBeAg negative HBV

Similarly to those with HBeAg positive disease, individuals with HBeAg negative disease were treated with PegIFNα for one year, and then treated with TDF thereafter (independent of disease activity), in line with NICE guidelines.[3] Whilst the aim of treatment for HBeAg positive individuals in long-term viral suppression and inducing HBeAg seroconversion, for HBeAg negative individuals, treatment aims for long-term viral suppression of HBV DNA.

It was assumed that in the first year of treatment with PegIFNα, 63% would achieve a virological response after one year, defined as HBV DNA <400 copies per ml, or 69 IU/ml.[7, 8] Furthermore, 2.8% of individuals with HBeAg negative disease achieved HBsAg seroconversion during one year of treatment with PegIFNα.[8]

All patients received TDF after one year of treatment with PegIFNα. For HBeAg negative patients, 1% received Emtricitabine alongside TDF.[6] Data from Marcellin *et al*. show long term virological control for HBeAg negative patients receiving TDF was 99% over five years, with 96% achieving virological response after 48 weeks.[6] We assumed an annual probability of 96% that individuals receiving TDF would achieve virological response (i.e. transitioning from ‘HBeAg active disease’ to ‘HBeAg inactive disease’). Individuals with HBeAg negative inactive disease, with or without cirrhosis, were assumed to remain on TDF after achieving virological control, as per treatment guidelines.[3]

During five years of follow-up from Marcellin et al, one individual had HBsAg loss, but it was not confirmed whether this individual achieved HBsAg seroconversion.[6] A conservative approach was taken and patients receiving TDF were unable to transition to the HBsAg seroconversion state, nor were those not receiving treatment.[6, 7] Similarly to HBeAg positive individuals, it was assumed that those on treatment had the same risk of disease progression as those with inactive disease. Data suggests that both HBeAg positive and negative patients receiving long-term TDF experience regression of fibrosis scores, including those with cirrhosis.[6] However, a conservative approach was taken, and the model did not allow for regression of cirrhosis to non-cirrhotic.

Appendix Figure 1: HBV Markov Model Structure


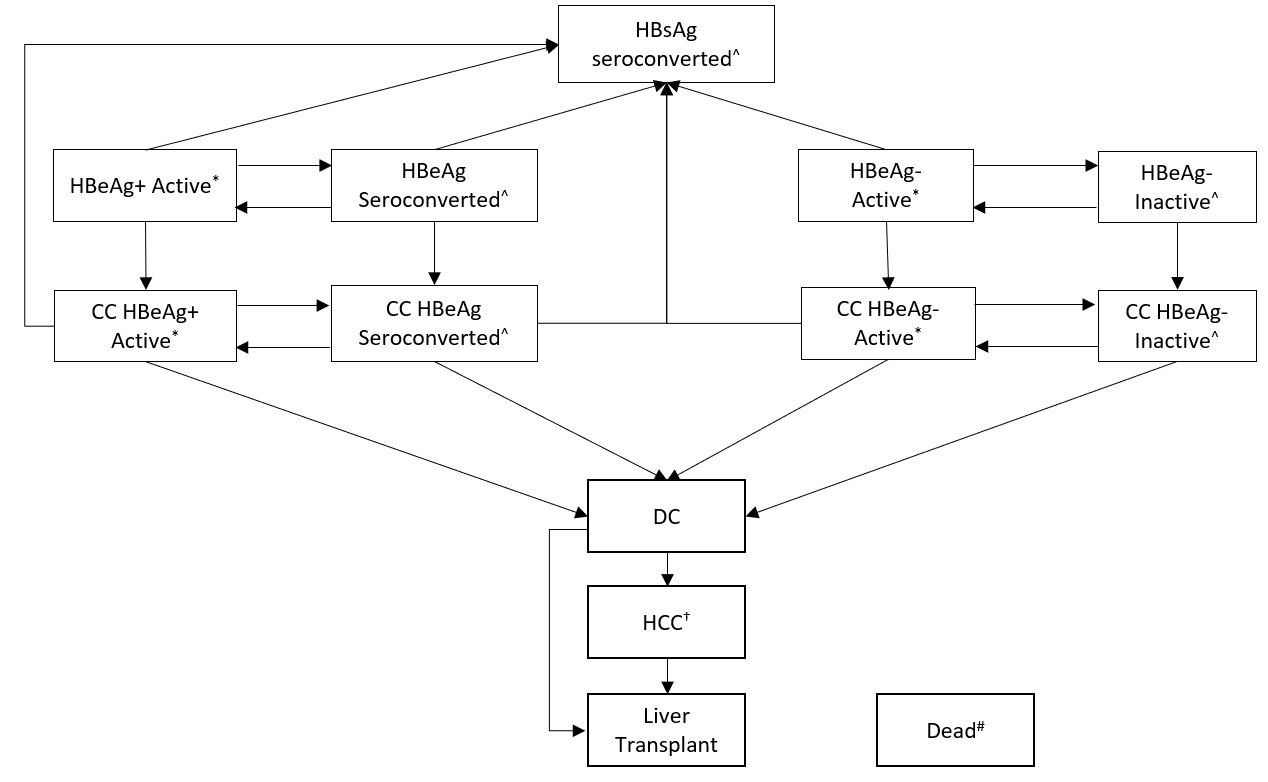


*Active health states include the following states: ‘undiagnosed’, ‘on treatment (year 1)’, ‘on treatment (>1 year)’, ‘diagnosed, not engaged in care’

^Seroconverted/inactive/HBsAg seroconverted health states include the following states: ‘undiagnosed’, ‘diagnosed, engaged in care’ ‘diagnosed, not engaged in care’

#Transition to death possible from all other health states as background (general population) mortality. Disease progression to death also occurs from active disease health states (without treatment), DC, HCC and Liver transplant health states.

†Transitions to HCC possible from all health states, except from liver transplant and dead states.

Appendix Figure 2: HCV Markov model structure


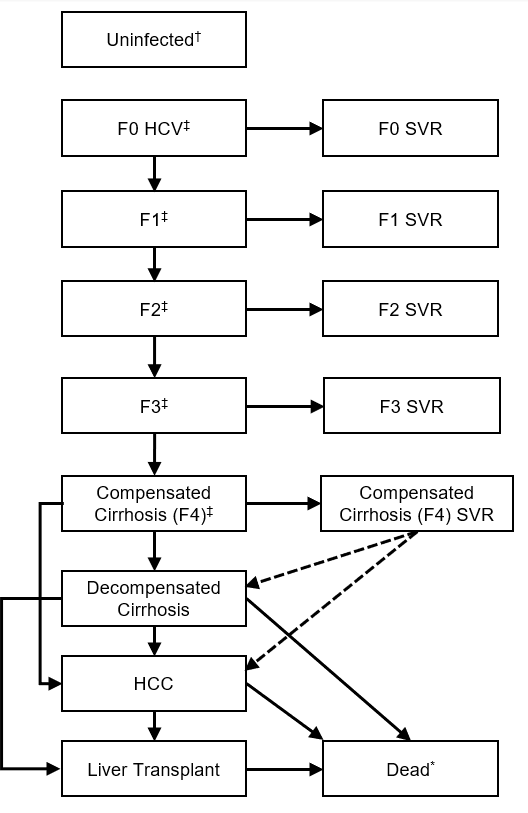


†Uninfected state contains the following health states: untested (RNA negative), untested (RNA positive) and tested.

‡Mild, moderate and compensated cirrhosis health states contain the following states: undiagnosed, on treatment, SVR (as shown above), non-SVR, and not engaged in care

* Transitions possible from all health states to dead, arrows show disease related mortality only.

Appendix Table 1: Other Model Probabilities

| **Base case probabilities** | **Mean Value** | **Distribution** | **Source** |
| --- | --- | --- | --- |
| **HBV Parameters** |  |  |  |
| Background testing probability (annual) | 2.50% | Beta (α=449446, β=17495668) | [9, 10] |
| Background test positivity | 0.98% | Beta (α=4399, β=445047) | [9] |
| Annual loss to follow up from treatment | 3.3% | Uniform (1.7%, 5%) | [2] |
| **HCV Parameters** |  |  |  |
| Background testing probability (annual) | 2.08% | Beta (α=372559, β=17572555) | [9, 10] |
| Background testing probability (annual) – PWID | 26.8% | Beta (α=1452, β=1674)^†^ | [11] |
| Standard mortality ratio for IDU (whilst currently injecting) | 7.8 | Normal (95% CI = 5.4-10.8) | [12] |
| Duration of injecting (years) | 11 | Uniform (6, 16) | [13] |
| Annual probability of reinfection amongst PWIDs | 19.34% | Beta (a = 15, b = 62)^‡^ | [14] |

^†^Distribution refers to two-year probability of testing, which is converted to an annual probability of testing using the following formula: *1-exp(log(1-probability)/2)*

^‡^Distribution refers to re-infection probability, whilst accounting for person years of follow-up amongst the cohort. The probability of reinfection was calculated using the following formula: *1-exp(-(0.1934/(69.79/77)))*

Appendix Table 2: Base case HBV transition probabilities for individuals entering the model with chronic HBV and HBeAg positive disease

| To: From: | HBsAg seroconverted | HBeAg+ active disease | HBeAg+ seroconverted | HBeAg+ CC active | HBeAg+ CC seroconverted | DC | HCC | LT  (first year) | LT  (≥1 year) | Dead |
| --- | --- | --- | --- | --- | --- | --- | --- | --- | --- | --- |
| HBsAg seroconverted | # | - | - | - | - | - | 0.00005 | - | - | - |
| HBeAg seroconverted | 0.02 | 0.03 | # | 0.01 | - | - | 0.001 | - | - | - |
| HBeAg+ active disease no treatment | 0.0175 | # | 0.05 | 0.05 | - | - | 0.005 | - | - | 0.0035 |
| HBeAg+ active disease, treatment with PegIFNα | 0.0295 | # | 0.32 | 0.01 | - | - | 0.0028 | - | - | - |
| HBeAg+ active disease, treatment with tenofovir | 0.018 | # | 0.0557 | 0.01 | - | - | 0.0028 | - | - | - |
| HBeAg+ compensated cirrhosis (CC) HBeAg seroconverted | 0.02 | - | - | 0.03 | # | 0.01 | 0.001 | - | - | - |
| HBeAg+ CC active – No treatment | - | - | - | # | 0.05 | 0.05 | 0.025 | - | - | 0.051 |
| HBeAg+ CC active – Treatment response with PegIFNα | 0.0295 | - | - | # | 0.32 | 0.01 | 0.0065 | - | - | - |
| HBeAg+ CC active – Treatment response with tenofovir | 0.018 | - | - | # | 0.0557 | 0.01 | 0.0065 | - | - | - |
| Decompensated cirrhosis (DC) | - | - | - | - | - | # | 0.025 | 0.03 | - | 0.39 |
| Hepatocellular carcinoma (HCC) | - | - | - | - | - | - | # | - | - | 0.56 |
| Liver Transplant (LT, first year) | - | - | - | - | - | - | - | # | - | 0.21 |
| LT (≥1 year post transplant) | - | - | - | - | - | - | - | - | # | 0.057 |

#represents the remainder of all transition probabilities (i.e. 1-all other transitions)

Transition probabilities are modelled probabilistically using a Dirichlet distribution, assuming a sample of size of 200, as performed in previous economic evaluations.

Sources: Shepherd 2006,[1] Lau 2005,[4] Marcellin 2013,[6] Kim 2015[15]

Appendix Table 3: Base case HBV transition probabilities for individuals entering the model with chronic HBV and HBeAg negative disease

| To: From: | HBsAg seroconverted | HBeAg- active disease | HBeAg- inactive disease | CC active disease | CC inactive disease | DC | HCC | LT  (first year) | LT  (≥1 year) | Dead |
| --- | --- | --- | --- | --- | --- | --- | --- | --- | --- | --- |
| HBsAg seroconverted | # | - | - | - | - | - | 0.00005 | - | - | - |
| HBeAg seroconverted | - | 0.029 | # | 0.01 | - | - | 0.005 | - | - | - |
| HBeAg- active disease no treatment | - | # | 0.015 | 0.09 | - | - | 0.005 | - | - | 0.0035 |
| HBeAg- active disease, treatment with PegIFNα | 0.0282 | # | 0.63 | 0.01 | - | - | 0.0028 | - | - | - |
| HBeAg- active disease, treatment with tenofovir | - | # | 0.96 | 0.01 | - | - | 0.0028 | - | - | - |
| HBeAg- compensated cirrhosis (CC) seroconverted | - | - | - | 0.029 | # | 0.01 | 0.005 | - | - | - |
| HBeAg- CC active – No treatment | - | - | - | # | - | 0.05 | 0.025 | - | - | 0.051 |
| HBeAg- CC active – Treatment response with PegIFNα | 0.0282 | - | - | # | 0.63 | 0.01 | 0.0065 | - | - | - |
| HBeAg- CC active – Treatment response with tenofovir | - | - | - | # | 0.96 | 0.01 | 0.0065 | - | - | - |
| Decompensated cirrhosis (DC) | - | - | - | - | - | # | 0.025 | 0.03 | - | 0.39 |
| Hepatocellular carcinoma (HCC) | - | - | - | - | - | - | # | - | - | 0.56 |
| Liver Transplant (LT, first year) | - | - | - | - | - | - | - | # | - | 0.21 |
| LT (≥1 year post transplant) | - | - | - | - | - | - | - | - | # | 0.057 |

#represents the remainder of all transition probabilities (i.e. 1-all other transitions)

Transition probabilities are modelled probabilistically using a Dirichlet distribution, assuming a sample of size of 200, as performed in previous economic evaluations.

Source: Shepherd 2006,[1] Marcellin 2004,[8] Marcellin 2013,[6] Kim 2015,[15] Takeda 2007.[16]

Appendix Table 4: HCV Transition probabilities

| **Base case probabilities** | **Mean** | **Distribution** | **Source** |
| --- | --- | --- | --- |
| ***Transition probabilities*** |  |  |  |
| F0 to F1 – non PWID | 0.107 | Beta(α=356.14, β=2972.28) | [17] |
| F1 to F2 – non PWID | 0.082 | Beta(α=328.12, β=3673.37) | [17] |
| F2 to F3 – non PWID | 0.117 | Beta(α=383.64, β=2895.35) | [17] |
| F3 to CC (F4) – non PWID | 0.116 | Beta(α=250.62, β=1909.88) | [17] |
| F0 to F1 – PWID | 0.109 | Beta(α=17.18, β=140.43) | [17] |
| F1 to F2 – PWID | 0.071 | Beta(α=37.1, β=485.43) | [17] |
| F2 to F3 – PWID | 0.121 | Beta(α=27.91, β=202.72) | [17] |
| F3 to CC (F4) – PWID | 0.194 | Beta(α=22.6, β=93.9) | [17] |
| CC to DC | 0.039 | Beta(α=14.617, β=360.1732) | [18] |
| CC to HCC | 0.014 | Beta(α=1.9326, β=136.1074) | [18] |
| CC SVR to DC (relative risk vs. non-SVR) | 0.07 | Lognormal(95% CI 0.03, 0.2) | [19] |
| CC SVR to HCC (relative risk vs. non-SVR) | 0.23 | Lognormal(95% CI 0.16, 0.35) | [20] |
| DC to HCC | 0.014 | Beta(α=1.9326, β=136.1074) | [18] |
| DC to liver transplant (LT) | 0.03 | Beta(α=6.5256, β=210.9945) | [18] |
| DC to death | 0.13 | Beta(α=147.03, β=983.97) | [18] |
| HCC to LT | 0.03 | Beta(α=6.5256, β=210.9945) | [18] |
| HCC to death | 0.43 | Beta(α=117.1033, β=155.23) | [18] |
| Post LT (first year) to death | 0.21 | Beta(α=16.2762, β=61.2294) | [18] |
| Post LT (after first year) to death | 0.057 | Beta(α=2.902, β=378.8825) | [18] |
| ***SVR related probabilities (post-DAA)*** |  |  |  |
| Mild / moderate | 0.928 | Beta(α=376, β=29) | [21] |
| CC | 0.908 | Beta(α=736, β=75) | [21] |
| Mild / moderate (retreatment) | 0.953 | Beta(α=82, β=4) | [22] |
| CC (retreatment) | 0.810 | Beta(α=44, β=10) | [22] |

Appendix Table 5: Health state costs

| **Costs (per year, except where noted)** | **Inflated cost** | **Cost year** | **Distribution** | **Source** |
| --- | --- | --- | --- | --- |
| **HBV Model** |  |  |  |  |
| HBsAg seroconverted | £0 | N/A | N/A | [1] |
| HBeAg seroconverted / inactive disease | £397 | 2002/03 | Gamma (k=25, θ= 10.6708) × PPI^†^ | [1] |
| HBeAg+ or HBeAg- active disease | £799 | 2002/03 | Gamma (k=25, θ=21.4992) × PPI^†^ | [1] |
| **HCV Model** |  |  |  |  |
| Mild (F0/F1) diagnosed | £205 | 2002/03 | Gamma (k=25.6995, θ=5.3698) × PPI^†^ | [18] |
| Moderate (F2/F3) diagnosed | £1,066 | 2002/03 | Gamma (k=88.8502, θ=8.0698) × PPI^†^ | [18] |
| Mild SVR | £257 | 2006/07 | Gamma (k=25, θ=8.08) × PPI^†^ | [23] |
| Moderate SVR | £314 | 2006/07 | Gamma (k=25, θ=9.88) × PPI^†^ | [23] |
| Cirrhosis SVR | £556 | 2006/07 | Gamma (k=25, θ=17.48) × PPI^†^ | [23] |
| **Both Models** |  |  |  |  |
| Cirrhosis (F4) diagnosed | £1,693 | 2002/03 | Gamma (k=24.2342, θ=46.9584) × PPI^†^ | [18] |
| Decompensated cirrhosis | £13,557 | 2002/03 | Gamma (k=36.0249, θ=253.1582) × PPI^†^ | [18] |
| Hepatocellular carcinoma | £12,081 | 2002/03 | Gamma (k=18.1081, θ=448.8045) × PPI^†^ | [18] |
| Liver transplant (per transplant) | £40,627 | 2002/03 | Gamma (k=89.7536, θ=304.5004) × PPI^†^ | [18] |
| Cost of care in year of liver transplant | £14,060 | 2002/03 | Gamma (k=13.7788, θ=686.4168) × PPI^†^ | [18] |
| Cost of care post liver transplant | £2,059 | 2002/03 | Gamma (k=15.2189, θ=91.0053) × PPI^†^ | [18] |

^†^PPI = NHS cost inflation index to 2019/20 (2002/03 = 1.487, 2006/07 = 1.272)

Appendix Table 6: Base case utility values

| **Health state** | **Mean** | **Distribution** | **Denominator (multiplicative method)** | **Relative utility^†^** | **Source** |
| --- | --- | --- | --- | --- | --- |
| **HCV Utility estimates** |  |  |  |  |  |
| Chronic HCV (F0-F3) | 0.751 | Beta (479.98, 159.14) | 0.869 | 0.864 | [24] |
| Chronic HCV (F0-F3) SVR increment | 0.035 | Beta (80.7, 2224.97) | N/A | N/A | [24] |
| **HBV Utility estimates** |  |  |  |  |  |
| HBsAg seroconverted | 1 | N/A | 1 | 1 | Assumption |
| Chronic HBV | 0.87 | Beta (1679.14, 250.91) | 0.93 | 0.935 | [25] |
| **Both models** |  |  |  |  |  |
| Cirrhosis | 0.671 | Beta (329.74, 161.68) | 0.869 | 0.772 | [24] |
| Decompensated cirrhosis | 0.602 | Beta (212.43, 140.44) | 0.869 | 0.693 | [24] |
| HCC | 0.662 | Beta (124.23, 63.43) | 0.869 | 0.762 | [24] |
| Liver transplant (after first year) | 0.657 | Beta (213.79, 111.61) | 0.869 | 0.756 | [24] |

^†^Relative utility was estimated by dividing the health state utility by the age-matched general population utility value. The relative utility value was then multiplied by the age-based general population utility, calculated in each annual cycle, using the following formula, derived from Ara and Brazier 2010[26]: *General Population, EQ-5D = 0.9508566 + 0.0212126*male – 0.0002587*age – 0.0000332*age^2*.

Appendix Table 7: One-way deterministic sensitivity analyses of HBV testing, in LTHT Leeds and GSTT London

|  | **ICER** | |
| --- | --- | --- |
| **Deterministic sensitivity analysis** | **LTHT, Leeds** | **GSTT, London** |
| Base case | £9,728 | £9,833 |
| Full time band 6 nurse for contacting patients^†^ | £9,930 | £9,945 |
| Proportion attending referral (50% for both settings)^‡^ | £13,598 | £13,064 |
| Proportion attending referral (90% for both settings)^‡^ | £8,090 | £8,592 |
| Treatment adherence - 6.7% disengage each year | £12,407 | £11,222 |
| Background rate of testing - 50% lower | £9,134 | £9,665 |
| Background rate of testing - 50% higher | £10,589 | £10,080 |
| Age of HBV+ case: 35 years | £9,575 | £8,997 |
| Age of HBV+ case: 55 years | £10,804 | £10,721 |
| Test costs equal to Leeds | - | £8,745 |

^†^Assumes cost to contact patients of £110.83 for LTHT in Leeds, and £81.66 for GSTT in London

^‡^The proportion of patients contacted and attending their referral in the base case is 69.2% for LTHT in Leeds, and 71.1% for GSTT in London.

Appendix Table 8: One-way deterministic sensitivity analyses of HCV testing, in LTHT Leeds and GSTT London

|  | **ICER** | |
| --- | --- | --- |
| **Deterministic sensitivity analysis** | **LTHT, Leeds** | **GSTT, London** |
| Base case | £7,177 | £12,387 |
| DAA Cost: £5000 | £4,512 | £10,106 |
| Full time band 6 nurse for contacting patients^†^ | £7,470 | £12,867 |
| Proportion attending referral - 50% lower (25.7% LTHT, 11.8% GSTT)^‡^ | £9,849 | £22,261 |
| Proportion attending referral - 50% higher (77.0% LTHT, 35.3% GSTT)^‡^ | £6,450 | £9,141 |
| PWID Proportion: 54.5% (equal to Parry *et al.*)[27] | £6,516 | £10,965 |
| Background rate of testing - 50% lower for non-PWID | £6,795 | £11,494 |
| Background rate of testing - 50% higher for non-PWID | £7,585 | £13,319 |
| Age of HCV+ case: 35 years | £5,864 | £8,782 |
| Age of HCV+ case: 55 years | £12,081 | £17,139 |
| Fibrosis scores derived from Leeds | - | £18,365 |
| Test costs equal to Leeds | - | £8,578 |

^†^Assumes cost to contact patients of £110.83 for LTHT in Leeds, and £81.66 for GSTT in London

^‡^The proportion of patients contacted and attending their referral in the base case is 51.4% for LTHT in Leeds, and 23.5% for GSTT in London.

Appendix Figure 3: Cost-effectiveness of HCV testing in LTHT Leeds, and GSTT London, across a range of DAA treatment costs

*
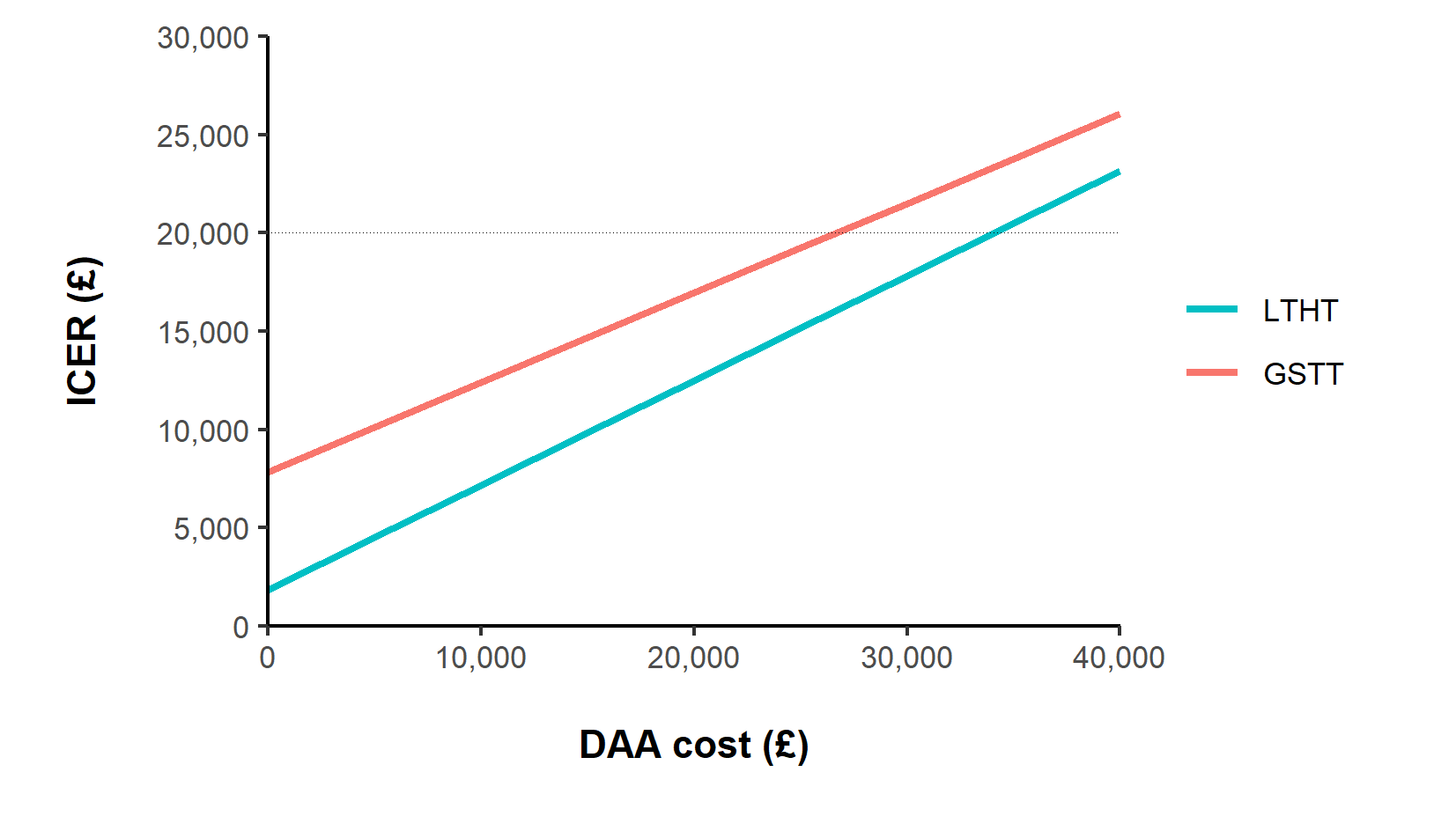
*

Appendix Table 9: Minimum prevalence at which HBV and HCV testing in the ED are 90% likely to be cost-effective in probabilistic analyses, for each setting, and across settings

| **Testing Strategy** | **Leeds** | **GSTT, London** | **Combined (both settings)** |
| --- | --- | --- | --- |
| HBV Testing | 0.55% | 0.6% | 0.6% |
| HCV Testing | 0.25% | 0.85% | 0.75% |

Appendix Table 10: Sensitivity analysis of budget impact of annual HBV and HCV testing assuming cost of contacting positive cases performed by full time band 6 nurse

| **Budget impact item** | **LTHT, Leeds^†^** | **GSTT, London^†^** |
| --- | --- | --- |
| **HBV Testing** |  |  |
| Testing costs | £49,673 | £174,690 |
| Cost of contacting positive cases^‡^ | £10,787 | £20,943 |
| Appointment costs for those engaged | £12,623 | £36,736 |
| **HBV Testing Total** | **£73,084** | **£232,369** |
| **HCV Testing** |  |  |
| Testing costs | £102,132 | £243,389 |
| Cost of contacting positive cases | £23,053 | £23,251 |
| Appointment costs for those engaged | £30,069 | £16,884 |
| Additional outreach costs (Find and Treat) | £9,002 | £8,517 |
| **HCV Testing Total** | **£164,256** | **£292,041** |

**^†^**Annual testing assumes 21,404 HBV and HCV tests per year in LTHT, Leeds, and 30,171 HBV and HCV tests per year in GSTT, London.

^‡^ Assumes cost per contacting each case of £110.83 for LTHT and £81.66 for GSTT.

# References

1. Shepherd J, et al., *Adefovir dipivoxil and pegylated interferon alfa-2a for the treatment of chronic hepatitis B: a systematic review and economic evaluation.* Health Technol Assess. **10**(28). (2006) DOI: <https://doi.org/10.3310/hta10280>.

2. Marcellin, P., et al., *Long-Term Treatment with Tenofovir Disoproxil Fumarate for Chronic Hepatitis B Infection Is Safe and Well Tolerated and Associated with Durable Virologic Response with No Detectable Resistance: 8 Year Results from Two Phase 3 Trials* 65th Annual Meeting of the American Association for the Study of Liver Diseases. (2014).

3. National Institute for Health and Care Excellence, *Hepatitis B (chronic): diagnosis and management (CG165)*. (2017). (2017)

4. Lau, G.K.K., et al., *Peginterferon Alfa-2a, Lamivudine, and the Combination for HBeAg-Positive Chronic Hepatitis B.* N Engl J Med. **352**(26): p. 2682-2695. (2005) DOI: 10.1056/NEJMoa043470.

5. National Institute for Health and Care Excellence, *Hepatitis B and C testing: people at risk of infection (PH43)*. (2013). (2013)

6. Marcellin, P., et al., *Regression of cirrhosis during treatment with tenofovir disoproxil fumarate for chronic hepatitis B: a 5-year open-label follow-up study.* Lancet. **381**(9865): p. 468-475. (2013) DOI: <https://doi.org/10.1016/S0140-6736(12)61425-1>.

7. European Association for the Study of the Liver, *EASL 2017 Clinical Practice Guidelines on the management of hepatitis B virus infection.* J Hepatol. **67**: p. 370-398. (2017).

8. Marcellin, P., et al., *Peginterferon Alfa-2a Alone, Lamivudine Alone, and the Two in Combination in Patients with HBeAg-Negative Chronic Hepatitis B.* N Engl J Med. **351**(12): p. 1206-1217. (2004) DOI: 10.1056/NEJMoa040431.

9. Public Health England, *Annual report from the sentinel surveillance of blood borne virus testing in England 2019*, in *Health Protection Report*. (2021). (2021)

10. Office for National Statistics, *England Population Estimates, mid-2019*. (2020). (2020)

11. Public Health England, *Unlinked Anonymous Monitoring (UAM) Survey of HIV and viral hepatitis among PWID: 2020 report*. (2020), Public Health England: London. (2020)

12. Hickman, M., et al., *Assessing IDU prevalence and health consequences (HCV, overdose and drug-related mortality) in a primary care trust: implications for public health action.* J Public Health (Oxf). **31**(3): p. 374-382. (2009) DOI: 10.1093/pubmed/fdp067.

13. Sweeting, M.J., et al., *Estimating the prevalence of ex-injecting drug use in the population.* Stat Methods Med Res. **18**(4): p. 381-395. (2008) DOI: 10.1177/0962280208094704.

14. Schulkind, J., et al., *High response and re-infection rates among people who inject drugs treated for hepatitis C in a community needle and syringe programme.* J Viral Hepat. **26**(5): p. 519-528. (2019) DOI: 10.1111/jvh.13035.

15. Kim, W.R., et al., *Impact of long-term tenofovir disoproxil fumarate on incidence of hepatocellular carcinoma in patients with chronic hepatitis B.* Cancer. **121**(20): p. 3631-3638. (2015) DOI: 10.1002/cncr.29537.

16. Takeda, A., et al., *A systematic review and economic evaluation of adefovir dipivoxil and pegylated interferon-alpha-2a for the treatment of chronic hepatitis B.* J Viral Hepat. **14**(2): p. 75-88. (2007) DOI: doi:10.1111/j.1365-2893.2006.00808.x.

17. Erman, A., et al., *Estimation of fibrosis progression rates for chronic hepatitis C: a systematic review and meta-analysis update.* BMJ Open. **9**(11): p. e027491. (2019) DOI: 10.1136/bmjopen-2018-027491.

18. Shepherd J, et al., *Interferon alfa (pegylated and non-pegylated) and ribavirin for the treatment of mild chronic hepatitis C: a systematic review and economic evaluation.* Health Technol Assess. **11**(11). (2006) DOI: <https://doi.org/10.3310/hta11110>.

19. Van der Meer, A.J., et al., *Association between sustained virological response and all-cause mortality among patients with chronic hepatitis c and advanced hepatic fibrosis.* JAMA. **308**(24): p. 2584-2593. (2012) DOI: 10.1001/jama.2012.144878.

20. Morgan, R.L., et al., *Eradication of hepatitis c virus infection and the development of hepatocellular carcinoma: A meta-analysis of observational studies.* Ann Intern Med. **158**(5): p. 329-337. (2013) DOI: 10.7326/0003-4819-158-5-201303050-00005.

21. Irving WL, McLauchlan J, and G. Foster, *Real world outcomes of DAA therapy for chronic hepatitis C virus infection in the HCV Research UK National cohort.* J Hepatol. **66**(1): p. S504. (2017) DOI: 10.1016/S0168-8278(17)31408-3.

22. Smith, D.A., et al., *Real world SOF/VEL/VOX retreatment outcomes and viral resistance analysis for HCV patients with prior failure to DAA therapy.* J Viral Hepat. **28**(9): p. 1256-1264. (2021) DOI: 10.1111/jvh.13549.

23. Grishchenko M, et al., *Cost-effectiveness of pegylated interferon and ribavirin for patients with chronic hepatitis C treated in routine clinical practice.* Int J Technol Assess Health Care. **25**(2): p. 171-180. (2009) DOI: <https://doi.org/10.1017/s0266462309090229>.

24. Saeed, Y.A., et al., *A Systematic Review and Meta-Analysis of Health Utilities in Patients With Chronic Hepatitis C.* Value in Health. **23**(1): p. 127-137. (2020) DOI: <https://doi.org/10.1016/j.jval.2019.07.005>.

25. Wong, W.W.L., et al., *Cost effectiveness of screening immigrants for hepatitis B.* Liver Int. **31**(8): p. 1179-1190. (2011) DOI: 10.1111/j.1478-3231.2011.02559.x.

26. Ara, R. and J.E. Brazier, *Populating an economic model with health state utility values: moving toward better practice.* Value Health. **13**(5): p. 509-18. (2010) DOI: 10.1111/j.1524-4733.2010.00700.x.

27. Parry, S., et al., *Implementing routine blood-borne virus testing for HCV, HBV and HIV at a London Emergency Department – uncovering the iceberg?* Epidemiol Infect **146**(8): p. 1026-1035. (2018) DOI: 10.1017/S0950268818000870.
